# Can chest high-resolution computed tomography findings diagnose pulmonary alveolar microlithiasis?*

**DOI:** 10.1590/0100-3984.2014.0123

**Published:** 2015

**Authors:** Flávia Angélica Ferreira Francisco, Rosana Souza Rodrigues, Miriam Menna Barreto, Dante Luiz Escuissato, Cesar Augusto Araujo Neto, Jorge Luiz Pereira e Silva, Claudio S. Silva, Bruno Hochhegger, Arthur Soares Souza Jr., Gláucia Zanetti, Edson Marchiori

**Affiliations:** 1 Fellow PhD degree, Program of Post-graduation in Radiology, Universidade Federal do Rio de Janeiro (UFRJ), Rio de Janeiro, RJ, Brazil.; 2 PhD, Professor, Program of Post-graduation in Radiology, Universidade Federal do Rio de Janeiro (UFRJ), Physician at the Service of Radiology, Hospital Universitário Clementino Fraga Filho da Universidade Federal do Rio de Janeiro (UFRJ) and Instituto D’Or de Pesquisa e Educação, Rio de Janeiro, RJ, Brazil.; 3 PhD, Professor, Program of Post-graduation in Radiology, Universidade Federal do Rio de Janeiro (UFRJ), Physician at the Service of Radiology, Hospital Universitário Clementino Fraga Filho da Universidade Federal do Rio de Janeiro (UFRJ), Rio de Janeiro, RJ, Brazil.; 4 PhD, Associate Professor of Radiology, Department of Medical Practice, Universidade Federal do Paraná (UFPR), Curitiba, PR, Brazil.; 5 PhDs, Associate Professors, Department of Medicine and Diagnostic Support, Universidade Federal da Bahia (UFBA), Salvador, BA, Brazil.; 6 MD, Radiology Department, Facultad de Medicina Clinica Alemana, Universidad del Desarrollo Santiago, Chile.; 7 PhD, Associate Professor of Imaging Diagnosis, Universidade Federal de Ciências da Saúde de Porto Alegre (UFCSPA), Porto Alegre, RS, Brazil.; 8 PhD, Professor, Faculdade de Medicina de São José do Rio Preto (Famerp), São José do Rio Preto, SP, Brazil.; 9 PhD, Professor, Program of Post-graduation in Radiology at Universidade Federal do Rio de Janeiro (UFRJ), Rio de Janeiro, Professor of Medical Practice, Faculdade de Medicina de Petrópolis, Petrópolis, RJ, Brazil.; 10 PhD, Full Professor Emeritus, Universidade Federal Fluminense (UFF), Niterói, RJ, Associate Professor, Universidade Federal do Rio de Janeiro (UFRJ), Rio de Janeiro, RJ, Brazil.

**Keywords:** Pulmonary alveolar microlithiasis, High-resolution computed tomography, Pulmonary calcifications

## Abstract

**Objective:**

The present study was aimed at retrospectively reviewing high-resolution computed tomography (HRCT) findings in patients with pulmonary alveolar microlithiasis in order to evaluate the frequency of tomographic findings and their distribution in the lung parenchyma.

**Materials and Methods:**

Thirteen patients (9 females and 4 males; age, 9 to 59 years; mean age, 34.5 years) were included in the present study. The HRCT images were independently evaluated by two observers whose decisions were made by consensus. The inclusion criterion was the presence of abnormalities typical of pulmonary alveolar microlithiasis at HRCT, which precludes lung biopsy. However, in 6 cases lung biopsy was performed.

**Results:**

Ground-glass opacities and small parenchymal nodules were the predominant tomographic findings, present in 100% of cases, followed by small subpleural nodules (92.3%), subpleural cysts (84.6%), subpleural linear calcifications (69.2%), crazy-paving pattern (69.2%), fissure nodularity (53.8%), calcification along interlobular septa (46.2%) and dense consolidation (46.2%).

**Conclusion:**

As regards distribution of the lesions, there was preferential involvement of the lower third of the lungs. No predominance of distribution in axial and anteroposterior directions was observed.

## Abstract (pt)

**Objetivo:**

Analisar, retrospectivamente, as tomografias computadorizadas de alta resolução (TCAR) de pacientes com microlitíase alveolar pulmonar, a fim de avaliar a frequência dos padrões tomográficos e sua distribuição no parênquima pulmonar.

**Materiais e Métodos:**

O estudo incluiu 13 pacientes (9 femininos e 4 masculinos) com idades variando de 9 a 59 anos (média de 34,5 anos). Os exames foram avaliados por dois observadores, de modo independente, e os casos discordantes, resolvidos por consenso. O critério de inclusão foi a presença de anormalidades na TCAR típicas de microlitíase alveolar pulmonar, o que prescinde a necessidade de exame histopatológico. Entretanto, em 6 casos foram realizadas biópsias pulmonares.

**Resultados:**

Os achados tomográficos predominantes foram opacidades em vidro fosco e pequenos nódulos parenquimatosos, presentes em 100% dos casos, seguidos de pequenos nódulos subpleurais (92,3%), cistos subpleurais (84,6%), calcificações lineares subpleurais (69,2%), padrão de pavimentação em mosaico (69,2%), fissura nodular (53,8%), calcificação ao longo dos septos interlobulares (46,2%) e consolidações densas (46,2%).

**Conclusão:**

Quanto à distribuição dos achados, houve acometimento preferencial dos terços inferiores. Não foi observado predomínio de distribuição dos sentidos axial e anteroposterior

## INTRODUCTION

Pulmonary alveolar microlithiasis (PAM) is a rare recessive autosomal disease characterized by intra-alveolar deposition of spherical calcified concretions (called calciferites, calcopherites or microliths), in the absence of any known calcium metabolism disorder. Most patients are asymptomatic at the moment of the diagnosis, and usually the disease is incidentally detected during routine exams, although it may be diagnosed during investigation on the familial history of the individual presenting with PAM^(1,2)^.

PAM is described in patients at different ages, with a long and progressive course that may lead to deterioration of the pulmonary function^(2)^. The affected individuals generally become symptomatic at the third or fourth decades of life. The disease may occur either sporadically or within the family, with 33% of the cases being hereditary following an autosomal recessive inheritance pattern^(1,2)^. PAM pathogenesis has been attributed to mutation in the gene (SLC34A2) that encodes a type IIb sodium-dependent phosphate transporter involved in the phosphate homeostasis in different organs including the lungs, which induces excess phosphate accumulation, favoring the development of microliths^(3)^.

Due to the marked dissociation between radiological features and clinical presentation of PAM, the diagnosis is sometimes based only on radiological findings^(1)^. High resolution computed tomography (HRCT) plays a very important role in the diagnosis of patients with suspected PAM, as HRCT findings are so characteristic that further diagnostic investigations such as histopathological analysis are generally unnecessary, particularly in patients whose families have other members with the disease^(1)^.

The present study was aimed at analyzing, by means of HRCT in 13 PAM patients, the most frequent tomographic findings and their distribution in the pulmonary parenchyma.

## MATERIALS AND METHODS

Retrospective and descriptive study of chest HRCTs of 13 PAM patients randomly selected by means of personal contacts with radiologists in ten different institutions in seven Brazilian states and one institution in Chile, in the period between 2007 and 2014. Nine of the 13 patients were women (69.2%) and 4 were men (30.8%), with ages ranging between 9 and 59 years (mean of 34.46 years) and median of 31 years. The study was approved by the Committee for Ethics in Research of Hospital Universitário Clementino Fraga Filho – Universidade Federal do Rio de Janeiro, Rio de Janeiro, RJ, Brazil.

The diagnoses were made from chest HRCTs based upon the observation of typical patterns of the disease. However, 6 patients were also submitted to lung biopsy, which allowed for histological confirmation in such cases.

Chest HRCTs were performed using different scanners, with the high resolution technique being utilized in all cases, with sections from the lung apices to the bases. Images were acquired using a slice thickness of 1 to 2 mm, with the patients in dorsal decubitus, during inspiration, utilizing a high spatial resolution filter for images reconstruction, with 10 mm increment, without intravenous administration of iodinated contrast agent. The images were obtained and reconstructed in a 512 × 512 matrix, digitized and photographed for evaluation of the lung fields with window width ranging between 1,000 and 1,500 Hounsfield units (HU) and level between –650 and –750 HU. For evaluation of the mediastinum, the width of the windows ranged from 350 to 400 HU, and the center between 40 and 60 HU. The analysis of the HRCT images was independently undertaken by two experienced thoracic radiologists, and the discordant results were resolved by consensus.

The following tomographic findings were analyzed: ground glass opacities, subpleural linear calcifications, small parenchymal nodules, calcifications along the interlobular septa, small subpleural nodules, nodular fissures, subpleural cysts, dense consolidations and crazy paving pattern.

The lesions distribution in the craniocaudal axis (upper, medium and lower) and in the axial axis (central, peripheral, without predominance), was also evaluated. The lung was divided along the craniocaudal axis from the apices to the level of the aortic arch; middle third, from the aortic arch up to 2 cm below the carina; and lower third, from 2 cm below the carina up to the costophrenic sinuses. The lesions were defined as central, when predominating in the internal third of the lungs, and peripheral, when predominating in the external thirds of the lungs, and also without predominance, when both locations occurred simultaneously.

Ground glass opacity was defined as increased pulmonary attenuation, with preserved bronchial and vascular margins.

Subpleural linear calcifications were described as such in those patients presenting with continuous linear calcified, juxtapleural opacities visible on the mediastinal window.

Small parenchymal nodules were characterized as round focal opacities measuring less than 10 mm in diameter.

The presence of calcifications along the interlobular septa was characterized in cases where linear, thin calcified opacities outlining the periphery of the secondary pulmonary lobe were visualized on the mediastinal windows.

Small, subpleural nodules were called small nodules along the pleural surface; and nodular fissures, as small nodules distributed along pulmonary fissures.

Consolidations were defined as being a homogeneous increase in the lung parenchymal attenuation coefficient, leading to loss of individualization of vascular structures and of airways walls. Consolidations were considered to be dense in cases where the density was greater than that of the soft tissues, as evaluated at the mediastinal window.

The crazy paving pattern was defined as the superimposition of ground glass opacities and interlobular septa thickening.

Subpleural cysts were defined as hypodense round structures measuring less than 10 mm in diameter, aligned next to the pleura.

The criteria for definition of such findings were those listed in the Fleishner Society Glossary of Terms for Thoracic Imaging^(4)^ and the utilized terminology is presented in “Terminologia para a descrição de tomografia computadorizada do tórax” (Terminology for Description of Chest Computed Tomography) of Colégio Brasileiro de Radiologia (Brazilian College of Radiology)^(5)^ and Comissão de Imagem da Sociedade Brasileira de Pneumologia e Tisiologia (Imaging Commission of Brazilian Society of Pneumology and Tisiology)^(6,7)^.

## RESULTS

**Figure 1:**
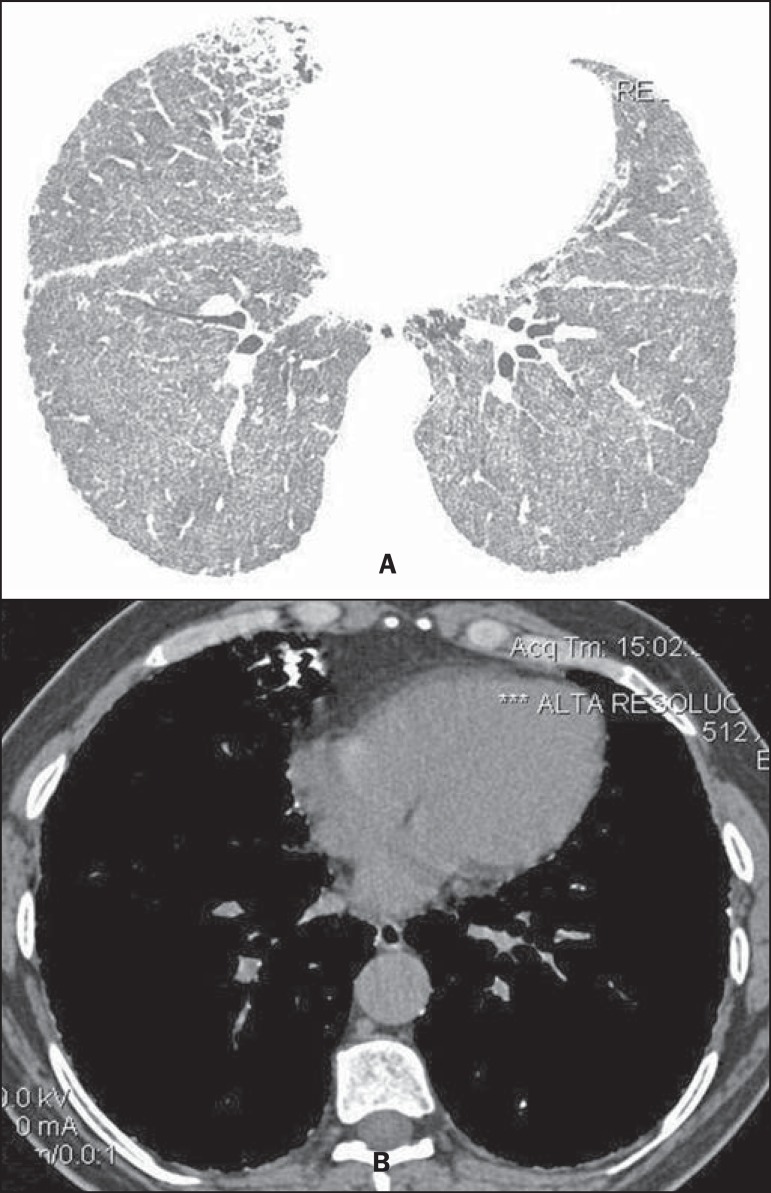
HRCT with lung windows setting shows (**A**) diffusely distributed ground glass opacities throughout the lungs, with consolidation in the middle lobe, besides nodular fissures. On mediastinal window (**B**) calcifications are observed in consolidations.

**Figure 2:**
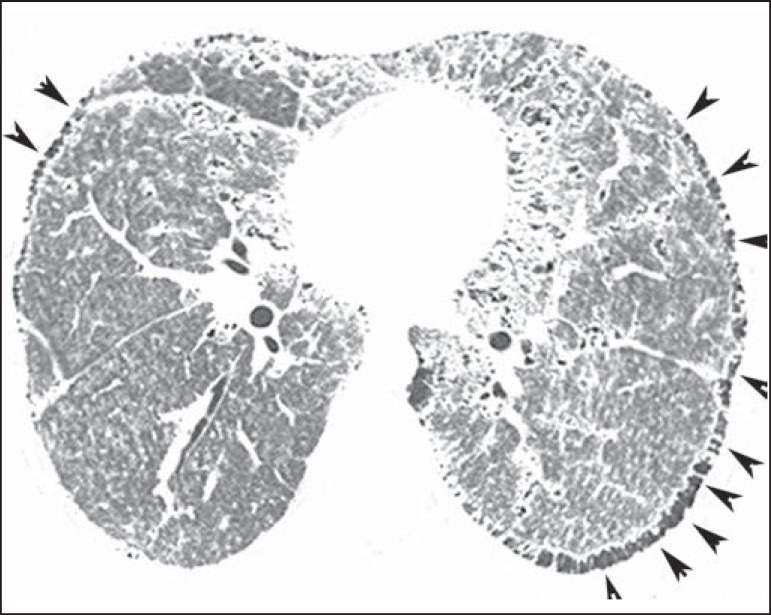
HRCT shows extensive, bilateral ground glass opacities diffusely distributed. Additionally, there are small subpleural cysts, more evident at the left (arrowheads).

**Figure 3:**
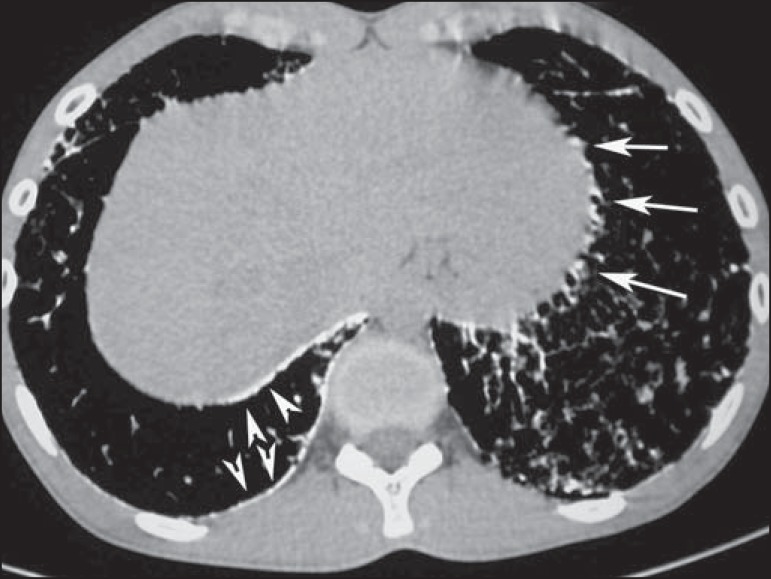
CT with mediastinal window demonstrating small calcified nodules with subpleural distribution (arrows) and subpleural linear calcifications (arrowheads).

**Figure 4:**
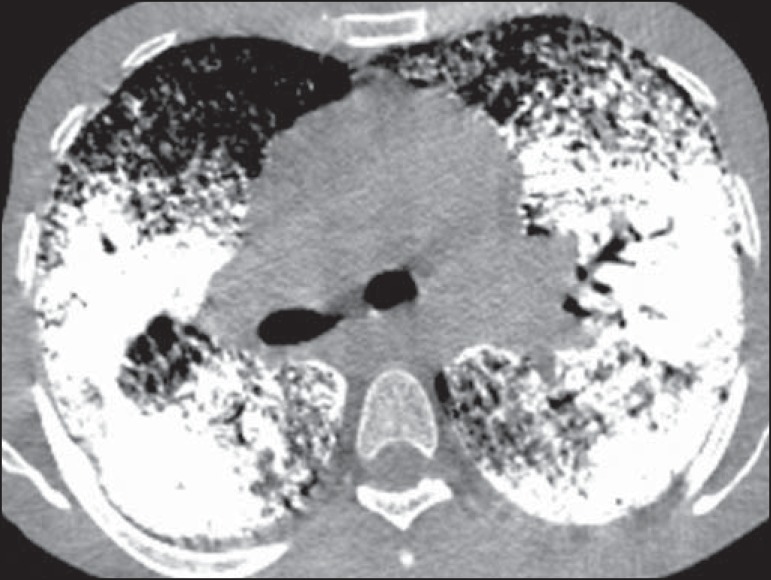
HRCT with mediastinal window demonstrating extensive consolidations with calcium density and intermingled air bronchograms bilaterally distributed throughout the lungs.

In decreasing order of frequency, the following tomographic findings were observed: ground glass opacities (n = 13; 100%) (Figure 1); small parenchymal nodules (n = 13; 100%); small subpleural nodules (n = 12; 92.3%); subpleural cysts (n = 11, 84.6%) (Figure 2); subpleural linear calcifications (n = 9; 69.2%) (Figure 3); crazy paving pattern (n = 9; 69.2%); nodular fissure (n = 7; 53.8%); calcification along interlobular septa (n = 6; 46.2%); and dense consoli-dations (n = 6; 46.2%). Intermingled air bronchograms were identified in the 6 cases (Figure 4). As regards findings distribution, the lower third of the lungs was predominantly affected. No predominance was observed in distribution of findings in the axial and anteroposterior axis.

## DISCUSSION

The imaging evaluation of the chest has been the subject of a series of recent publications in the Brazilian radiological literature^(8-18)^. With respect to PAM, studies analyzing large samples have not detected any gender predominance^(2)^. In the present study sample, 9 patients (69.2%) were women and 4 (30.8%) were men. Such difference is possibly due to the limited number of studied patients.

As regards age at PAM diagnosis, studies demonstrated that the disease has already been described in patients of different age groups, from children under one year of age to elderly patients^(2,19)^. In the literature, it was observed that 35.8% of the patients were less than 20 years old, while 88.2% were less than 50 years old^(2)^. In the present study, the ages ranged between 9 and 59 years, with a mean age of 34.5 years and median of 31 years, therefore our data was very similar to those found in the literature.

It is difficult to establish the age of PAM onset, as the time when it is diagnosed does not necessarily reflect the period when the disease started, because an important characteristic of this disease is the clinico-radiological dissociation, meaning that symptoms are scarce in contrast with the imaging findings^(20)^. Thus, many cases are diagnosed at imaging studies performed for other reasons, even in asymptomatic patients or, as the patient is symptomatic, the disease is diagnosed at more advanced stages^(2,21)^.

The affected individuals generally become symptomatic at the third or fourth decades of life. With the progression of the disease, which generally takes 10 to 20 years, cyanosis and digital clubbing are commonly observed. Dyspnea is the most frequent symptom, followed by dry coughing, chest pain, hemoptysis and asthenia, besides possible occurrence of pneumothorax^(2,10,22-24)^. Progressive deterioration of the pulmonary function may occur in adults, and death generally occurs in mid-adulthood because of respiratory failure with *cor pulmonale*^(14)^. On the other hand, cases of either static or very indolent disease have already been described^(2)^.

In approximately one third of the cases, PAM presents with a familial history, as it is a disease with recessive autosomal inheritance, indicating the importance of genetic factors in its genesis^(1)^. All PAM cases, either those of familial occurrence or the other two-thirds in the form of sporadic acquisition, present with mutation in the SLC34A2 gene, that is involved in the pathogenesis of the disease^(3,24,25)^.

Histopathological analysis reveals the presence of intraalveolar microliths. Sometimes, chronic inflammatory cells are present, and may induce interstitial fibrosis^(26)^. In some cases, microliths may be found in the sputum or in bronchoalveolar lavage, which can contain alveolar macrophages either with or without carbon particles^(27)^. With basis on HRCT findings, it is possible to confirm the diagnosis of PAM, many times avoiding lung biopsy ^(2,28-31)^. In the present study, the diagnosis was based on typical chest HRCT findings. However, 6 patients were submitted to open lung biopsy, thus allowing histopathological confirmation. Once a given patient is diagnosed with PAM, family members should be screened by means of chest radiography, and parents should be advised that future daughters and sons are also at risk of developing the disease^(28)^.

The most frequent findings reported in literature include diffuse ground glass attenuation and subpleural linear calcifications. Other findings include: small parenchymal nodules; calcifications along interlobular septa, sometimes determining the crazy paving pattern; small subpleural nodules, nodular fissures, subpleural cysts and dense consolidations^(29)^.

In the literature, ground glass opacities are reported as common findings, and probably occur due to the presence of small microliths in the air spaces^(29,32)^. In the present study, this was one of the main findings, present in all 13 cases (100%).

Small parenchymal nodules were also identified in all 13 patients (100%). At HRCT, small nodules at HRCT generally correspond to dense micronodules (< 3 mm), diffusely distributed throughout the parenchyma, making the lungs hypotransparent and characterizing the pattern described by some authors as “sandstorm”, that is considered as being typical of the disease. At HRCT, many times it is impossible to define the calcium density in the nodules, due to their small dimensions. However, when the microliths converge to form parenchymal consolidation, the calcium density can be better characterized, and is higher than soft tissues^(23,29)^.

Small subpleural nodules were observed in 12 of the cases (92.3%) and nodular fissures were observed in 7 patients (53.8%). These changes represent the accumulation of intra-alveolar microliths in the periphery of secondary lung lobes, adjacent to pleural surfaces, determining the feature of pseudopleural calcifications^(33,34)^.

Subpleural cysts represent another finding frequently reported in studies relying on HRCT. The small thin-walled cysts located in the subpleural spaces may determine the presence of a radiolucent line lying between the calcified parenchyma and the adjacent ribs, which is described in chest radiography as the “black pleural sign”^(29,35)^. In the present study, subpleural cysts were observed in 11 patients (84.6%).

Linear subpleural calcifications were found in 9 of the patients (69.2%). This finding, in spite of being commonly seen at computed tomography in patients with PAM, is controversial in the literature. Although their description by some authors as pleural calcifications, there is no study reporting histopathological confirmation of pleural calcification. For that reason, the best explanation seems to be that such a finding is a consequence of the accumulation of intra-alveolar microliths in the periphery of secondary lung lobes, adjacent to the pleural surfaces, thus producing the pseudopleural calcification feature^(33,34)^.

Calcifications along interlobular septa were observed in 6 patients, corresponding to 46.2% of the sample. Such a pattern occurs due to the deposition of microliths in the periphery of the secondary lung lobe, as there is no histopathological confirmation of calcification in interlobular septa or interstitial involvement at early stages of the disease^(29,36)^.

Crazy paving pattern was observed in 9 patients, representing 69.2% of the sample. This pattern is defined as areas of ground glass attenuation associated with interlobular septa thickening. It may also be observed on images at mediastinal windows due to the presence of calcifications along interlobular septa. There are no reports in literature about any other disease with similar tomographic manifestations. Thus, such HRCT findings are considered to be very specific and even pathognomonic of PAM^(29,36)^.

Confluence of small nodules may form areas of consolidation in the parenchyma, which, due to the presence of calcium, may have higher density than soft tissues. Dense consolidations predominantly occur in the cardiac margins, as well as in the lower and posterior regions of the lungs, tending to be symmetrical^(23,34,35)^. Dense consolidations with intermingled air bronchograms were found in 6 patients (46.2%) in the present study. Therefore, PAM should be considered as a differential diagnosis for the dense consolidation pattern^(23,29,34,35)^, together with amiodarone pulmonary toxicity^(37)^, metastatic pulmonary calcification^(38-41)^, silicoproteinosis^(42,43)^, talcosis^(44-46)^ and amyloidosis^(47-49)^.

In PAM, lesions may be limited to determined areas or present with diffuse and symmetrical distribution throughout the lungs^(21,23,29,34,35)^. Calcium deposition in the alveoli generally starts in the lower lobes and takes the entire lung over a period of years, with evolvement to the middle thirds and then to the upper portions of the lungs^(2)^. The tomographic alterations are predominant in the lower and posterior portions of the lungs^(29,34-36)^. Additionally, the central portions of the lungs are more affected than the peripheral portions^(35)^. In the present study, all patients presented with bilateral distribution of tomographic alterations, without laterality predominance. Also, parenchymal lesions compatible with PAM were found in all lung lobes.

Studies describing tomographic findings in PAM are restricted either to isolated case reports, or to case reports with a small number of patients. There are no available studies evaluating radiographic findings in PAM with a large series of patients.

Our study has some limitations. The study was retrospective and the analysis was cross-sectional, without evaluating clinical and evolutive data. The techniques of HRCT varied according to the protocols from each institution involved in the study. However, such factors did not impair the analysis of imaging features, which was actually the aim of the present study. Despite these limitations, this is the largest reported series of HRCT findings in patients with PAM.

In conclusion, the most frequent tomographic findings were diffuse ground glass attenuation and small parenchymal nodules followed by small subpleural nodules, subpleural cysts, subpleural linear calcifications, crazy paving pattern, nodular fissure, calcification along interlobular septa and dense consolidations. In general, the findings predominated in the lower third of the lungs. The typical HRCT findings in PAM are so characteristic that, when present, can rule out the need for lung biopsy.
